# Standardization of health outcomes assessment for depression and anxiety: recommendations from the ICHOM Depression and Anxiety Working Group

**DOI:** 10.1007/s11136-017-1659-5

**Published:** 2017-08-07

**Authors:** Alexander Obbarius, Lisa van Maasakkers, Lee Baer, David M. Clark, Anne G. Crocker, Edwin de Beurs, Paul M. G. Emmelkamp, Toshi A. Furukawa, Erik Hedman-Lagerlöf, Maria Kangas, Lucie Langford, Alain Lesage, Doris M. Mwesigire, Sandra Nolte, Vikram Patel, Paul A. Pilkonis, Harold A. Pincus, Roberta A. Reis, Graciela Rojas, Cathy Sherbourne, Dave Smithson, Caleb Stowell, Kelly Woolaway-Bickel, Matthias Rose

**Affiliations:** 1 Department of Psychosomatic Medicine, Center for Internal Medicine and Dermatology, Charité – Universitätsmedizin Berlin, Freie Universität Berlin, Humboldt-Universität zu Berlin, and Berlin Institute of Health, Charitéplatz 1, 10117 Berlin, Germany; 2 International Consortium for Health Outcomes Measurement (ICHOM), 14 Arrow St., Ste. #11, Cambridge, MA 02138 USA; 3 Harvard Medical School, 25 Shattuck Street, Boston, MA 02115 USA; 4 Massachusetts General Hospital, 55 Fruit Street, Boston, MA 02114 USA; 5 Department of Experimental Psychology, University of Oxford, Tinbergen Building, South Parks Road, Oxford, OX1 3UD UK; 6 Institut Philippe-Pinel de Montréal, 10905 Henri-Bourassa Est, Montréal, QC H1C 1H1 Canada; 7 Department of Psychiatry, University of Montréal, Pavillon Roger-Gaudry, C.P., 6128 succursale Centre-ville, Montréal, QC H3C 3J Canada; 8 Stichting Benchmark GGZ, Rembrandtlaan 46, 3723 BK Bilthoven, The Netherlands; 9 Department of Clinical Psychology, Leiden University, Rapenburg 70, 2311 EZ Leiden, The Netherlands; 10 Netherlands Institute for Advanced Study, Meijboomlaan 1, 2242 PR Wassenaar, The Netherlands; 11 The Center for Social and Humanities Research, King Abdulaziz University, Jeddah, 21589 Saudi Arabia; 12 School of Public Health, Kyoto University Graduate School of Medicine, Yoshida Konoecho, Sakyo-ku, Kyoto, 606-8501 Japan; 13 Department of Clinical Neuroscience, Karolinska Institutet, Nobels väg 9, 17177 Stockholm, Sweden; 14 Gustavsberg Primary Care Clinic, Stockholm County Council, Odelbergs väg 19, 13440 Gustavsberg, Sweden; 15 Centre for Emotional Health, Department of Psychology, Macquarie University, Building C3A, Level 7, Sydney, NSW 2109 Australia; 16 Ottawa, ON Canada; 17 Research centre, Institut universitaire en santé mentale de Montréal, 7401 rue Hochelaga, Montréal, QC H1N 3M5 Canada; 18 Makerere College of Health Sciences, Makerere University, P.O. Box 7072, Kampala, Uganda; 19 Population Health Strategic Research Centre, School of Health and Social Development, Deakin University, 221 Burwood Highway, Burwood, VIC 3125 Australia; 20 Department of Global Health and Social Medicine, Harvard Medical School, 641 Huntington Avenue, Boston, 02115 MA USA; 21 Department of Psychiatry, University of Pittsburgh School of Medicine, Thomas Detre Hall, 3811 O’Hara Street, Pittsburgh, PA 15213 USA; 22 Department of Psychiatry, Columbia University, New York Presbyterian Hospital, 630 West 168th Street, New York, NY 10032 USA; 23 The RAND Corporation, 1776 Main Street, Santa Monica, CA 90407-2138 USA; 24 Federal University of Rio Grande do Sul, Grupo CNPq - GPEMSA - USP/São Paulo, Av. Paulo Gama, 110 - Bairro Farroupilha, Porto Alegre, Rio Grande do Sul Brazil; 25 Clinical Hospital, Department of Psychiatry and Mental Health, University of Chile, Av. Libertador Bernardo O’Higgins 1058, Santiago de Chile, Chile; 26 Anxiety UK, Zion Community Centre, 339 Stretford Road, Hulme, Manchester M15 4ZY UK; 27 Office of the U.S. Army Surgeon General, 7700 Arlington Blvd., Room 3SW116A, Falls Church, VA 22042 USA; 28 Department of Quantitative Health Sciences, Medical School, University of Massachusetts, 368 Plantation Street, Worcester, MA 01605 USA

**Keywords:** Depression, Anxiety, Patient-reported outcomes, Health-related quality of life, Standardization, Outcome Set

## Abstract

**Purpose:**

National initiatives, such as the UK Improving Access to Psychological Therapies program (IAPT), demonstrate the feasibility of conducting empirical mental health assessments on a large scale, and similar initiatives exist in other countries. However, there is a lack of international consensus on which outcome domains are most salient to monitor treatment progress and how they should be measured. The aim of this project was to propose (1) an essential set of outcome domains relevant across countries and cultures, (2) a set of easily accessible patient-reported instruments, and (3) a psychometric approach to make scores from different instruments comparable.

**Methods:**

Twenty-four experts, including ten health outcomes researchers, ten clinical experts from all continents, two patient advocates, and two ICHOM coordinators worked for seven months in a consensus building exercise to develop recommendations based on existing evidence using a structured consensus-driven modified Delphi technique.

**Results:**

The group proposes to combine an assessment of potential outcome predictors at baseline (47 items: demographics, functional, clinical status, etc.), with repeated assessments of disease-specific symptoms during the treatment process (19 items: symptoms, side effects, etc.), and a comprehensive annual assessment of broader treatment outcomes (45 items: remission, absenteeism, etc.). Further, it is suggested reporting disease-specific symptoms for depression and anxiety on a standardized metric to increase comparability with other legacy instruments. All recommended instruments are provided online (www.ichom.org).

**Conclusion:**

An international standard of health outcomes assessment has the potential to improve clinical decision making, enhance health care for the benefit of patients, and facilitate scientific knowledge.

**Electronic supplementary material:**

The online version of this article (doi:10.1007/s11136-017-1659-5) contains supplementary material, which is available to authorized users.

## Introduction

Treatment of depression and anxiety disorders remains one of today’s most important health challenges. Combined, these two conditions represent the most years lived with disability of any disease [1]. Their direct treatment and indirect impact on other conditions contributes to a substantial portion of health care spending [2]. According to the most recent data available, depression in the United States alone costs society $210 billion per year, including direct medical costs (45%), suicide-related mortality costs (5%), and workplace costs (50%) [3].

A variety of treatment options have been proven effective in reducing symptom burden and improving functioning for patients with depression or anxiety [4]. These include several types and combinations of psychological interventions and antidepressant medications [5]. Although the general effectiveness of these treatments has been established, the questions of what works for whom and how to sequence and combine treatments remain to be addressed [6].

There are many well-validated health outcome assessments available to monitor the treatment process of mental health conditions [7]. However, utilization of empirical evidence in clinical practice to inform clinical decision making is still a rare occurrence.

There are a number of reasons why the empirical assessment of mental health domains is less common compared to the assessment of biomedical markers. Several methodological issues have been discussed, including insufficient measurement precision, limited measurement range, high respondent burden, inadequate physician reports, and the impracticality of using paper-and-pencil assessments within daily clinical routine [8]. Another important issue is that for many of the most relevant mental health domains there are several competing tools, and even if the same constructs are measured results from different instruments are difficult to compare [9]. Like in many other fields, lack of standardization seriously hinders communication among patients, practitioners, and scientists.

To date, the most comprehensive effort to initiate standardized outcome assessments for the treatment of mental health disorders has come from the United Kingdom (“Improving Access to Psychological Therapies” (IAPT)) [10]. Routine collection of patient-reported outcomes (PRO) was coupled to a new program of expanding access to psychotherapists [11]. The success of the program (63.7% achieved reliable improvement or recovery) was celebrated, and has supported the case for its funding and led to similar initiatives in other health systems [12–14]. Unfortunately, as outcome monitoring initiatives proliferate, no consensus exists as to which measures to include in such programs and many are now developing without awareness of existing global practices. Lack of data standards between programs hinders comparisons of program effectiveness or opportunities for data aggregation and research.

To address this need for a consolidated recommendation on what outcomes are essential to track for patients with depression and anxiety, we convened an international, multi-disciplinary Working Group under the leadership of the International Consortium for Health Outcomes Measurement (ICHOM).

## Method

### The Working Group

A Working Group was organized by ICHOM (www.ichom.org), a non-profit organization focused on the development of standardized datasets of outcomes and case-mix factors for use in clinical practice. Working Group members were selected by purposive sampling [15] based on their expertise with the aim of representing a wide clinical, scientific and cultural background. Members (*n* = 24) included patient representatives (LL, DS), measurement experts (EdB, EH, SN, PP, CS, MR), clinical (LB, TF, DM, RR, GR, KWB), social and public health researchers (AC, DC, PE, MK, AL, VP), clinicians (AO, LB, TF, MK, AL, DM, HP, RR, GR, MR) and coordinators (LvM, CSt). The final group included members from twelve countries: Australia, Brazil, Canada, Chile, Germany, India, Japan, the Netherlands, Sweden, Uganda, the United Kingdom, and the United States. Patient representatives participated in the development process of the standard set in every step, with equal voting rights, and contributed actively to the discussion.

### Development of the standard set

**Fig. 1:**
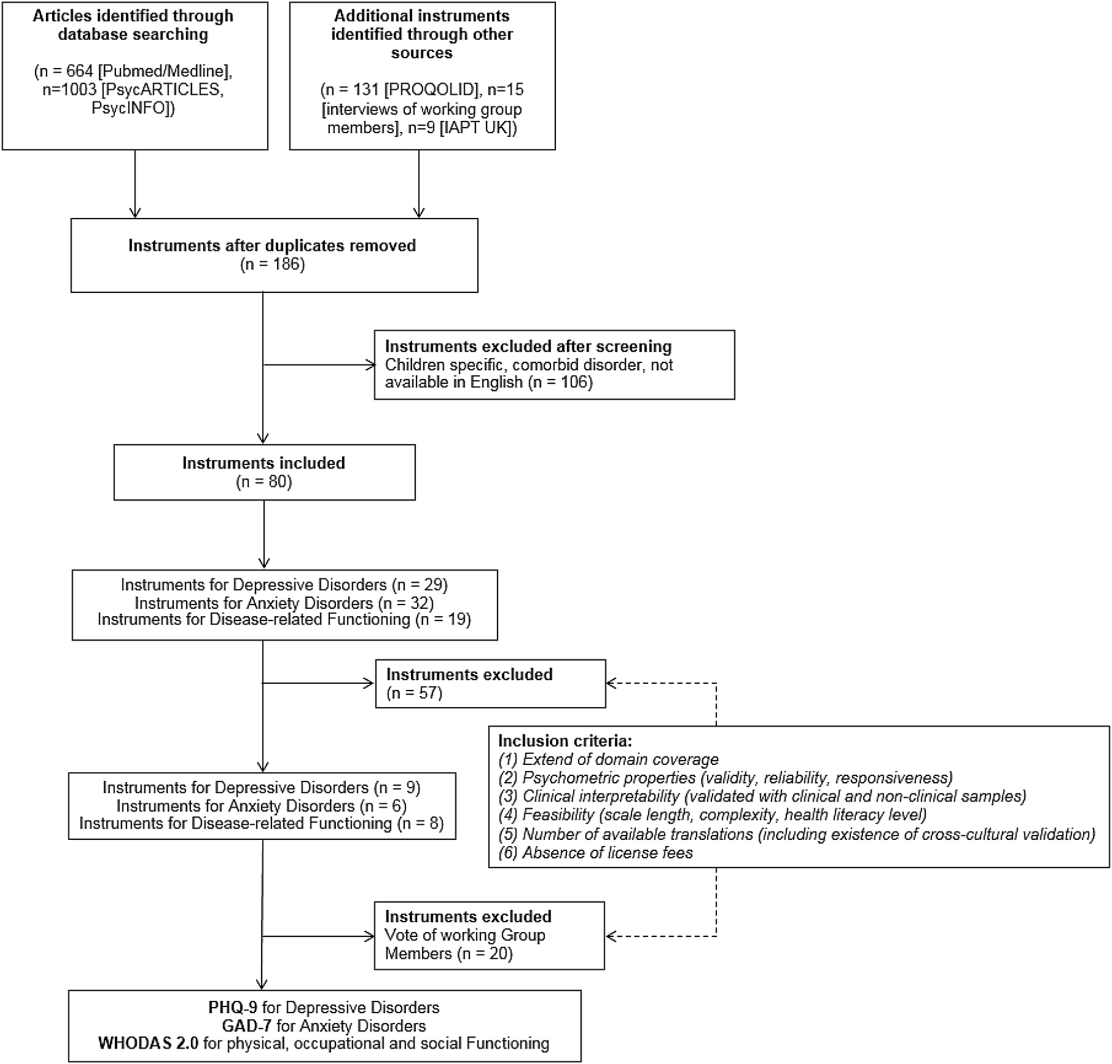
Search strategy and selection process for instruments considered for the D+A standard set (modified PRISMA flow diagram). Stepwise selection based on literature review, monthly teleconference calls, and subsequent online surveys. Initial search term for scientific databases: “(depress* [TITLE] OR anxiety [TITLE] OR PTSD [TITLE] OR post-traumatic stress disorder [TITLE] OR dysthymia [TITLE] OR GAD [TITLE] OR SAD [TITLE] OR agoraphobia [TITLE] OR panic [TITLE] OR obsessive compulsive [TITLE] OR OCD [TITLE]) AND (instrument [TITLE] OR patient-reported outcome [TITLE] OR questionnaire [TITLE])”. IAPT UK = Improving Access to Psychological Therapies program by the National Institute of Health in the United Kingdom; PHQ-9 = Patient Health Questionnaire 9-item version; GAD-7 = PHQ module for assessment of General anxiety disorder, 7-items; WHODAS = WHO Disability Assessment Schedule

A structured consensus-driven modified Delphi technique was used to develop the ICHOM standard set. The Delphi approach is an iterative, multistage process with the aim of transforming opinion into group consensus [16]. The technique was developed by ICHOM and successfully applied to create outcome standards for a growing number of health conditions (www.ichom.org) [17–23]. Over a period of eleven months, the Working Group met monthly by teleconference. Preparation of the meetings and surveys, guided by the ICHOM framework, followed a pre-defined set of activities: (1) prioritizing outcome domains, (2) selecting outcome measures, (3) prioritizing case-mix domains, and (4) selecting case-mix definitions. Prioritization of outcome domains and case-mix variables was carried out by allocating all variables to the outcome measures hierarchy developed by Porter [24]. In preparation of the teleconference calls, a comprehensive literature search using common databases (PubMed, EMBASE, Medline, PsycINFO) and a specific database for clinical outcome assessments (www.proqolid.org) was conducted for each outcome domain or case-mix factor, augmented by interviews with the patient representatives in the Working Group and selected experts (see Fig. 1 for a detailed search strategy, see Online Appendix 1 for a list of all instruments found, see “Results” section for a summary). During the teleconferences, the collated evidence was presented. Following each teleconference, the discussion content (qualitative data) was collated into online surveys (quantitative data). Working Group members were then asked to submit their feedback; final votes were carried out via an anonymous web questionnaire. Content was included if a two-third majority vote (66%) was reached, items rated below 50% were excluded, results between 50 and 66% were subject to further discussion in subsequent teleconferences and re-voted upon until consensus for in- or exclusion was reached. Results were fed back to the group in summarized form. Within eleven months of the duration of the project, seven surveys were conducted with response rates between 70 and 100%. Online surveys were compiled using Qualtrics^®^ online survey platform (www.qualtrics.com). The final standard set was approved by all members of the Working Group. Explanation of the consensus process (Online Appendix 2) and voting results (Online Appendix 3–7) are provided as online supplements.

In selecting measures for prioritized domains, available measurement tools covering the selected domains were reviewed. If there were no validated tools for prioritized case-mix variables, ad hoc items were generated based on existing instruments (IAPT UK [10], Canadian Community Health Survey [25]) modified to be appropriate for low health literacy levels [26]. This was not the case for outcome instruments (i.e., scales) but only for 13 single items included for case-mix adjustment collecting information about the patients’ medical history, such as the duration of symptoms or prior episodes of their disease (Table 1). Pre-defined inclusion criteria for instruments or single items comprised the following criteria: (1) extent of domain coverage (extent to which the instrument or item covers the a priori defined domains, for example, whether a questionnaire or set of questionnaires measuring functioning completely covered physical, social and occupational functioning or only partially), (2) psychometric properties (validity, reliability, responsiveness), (3) clinical interpretability (if instrument was validated with clinical and non-clinical samples), (4) feasibility (scale length, complexity, health literacy level), (5) number of available translations (including existence of cross-cultural validation), and (6) absence of license fees. In addition, instruments had to be available at least in English, and instruments and items had to be applicable for patients from age 14 and above. The selection process for the outcome measures is further illustrated in Fig. 1.

**Table 1:** Adapted or newly developed items in the standard set

#	Variable	Item	Response options
1	Age	What is your date of birth?	Date
2	Sex	Please indicate your sex at birth	Male, female, do not want to answer
3	Educational level	Please indicate highest level of schooling completed	ISCED 1997, Country specific
4	Living status	Which statement best describes your living arrangements?	(a) With partner/spouse/family/friends (b) Alone (c) Nursing home/hospital/long-term care home (d) Other
5	Work status	What is your work status?	(a) Unable to work (due to a condition other than depression or anxiety) (b) Unable to work (due to depression or anxiety) (c) Not working by choice (student, retired, homemaker) (d) Working part-time (e) Seeking employment (I consider myself able to work but cannot find a job) (f) Working full-time
6	Prior episodes of depression/anxiety	Did you experience similar episodes of depression or anxiety before in your life?	(a) This is my first episode (b) I had one similar episode before the current one (c) I had several similar episodes before the current one (d) My symptoms of depression do not occur in episodes
7	Duration of symptoms	How many months have you been experiencing symptoms of depression/anxiety?	# Of months
8	Prior/current treatment	During the last year, did you receive any of the following treatments for depression/anxiety? Response for each: medication, psychological treatment, other	(a) No (b) 1–3 months (c) 3–6 months (d) more than 6 months
9	Outcome expectancy	How successful do you think your current therapy will be in reducing your symptoms?	(a) Not at all successful (b) Somewhat successful (c) Moderately successful (d) Very successful
10	Medication side effects	Did you experience medication side effects? If Yes, please indicate which side effects you have experienced:	yes/no (a) Weight gain (b) Sexual dysfunction (c) Sleep disturbances (d) Dry mouth (e) Drowsiness/sedation (f) Cardiovascular side effects (e.g. palpitations) (g) Gastrointestinal side effects (e.g. diarrhea, nausea, vomiting) (h) Other:
11	Absenteeism	How many working days have you missed within the last month due to illness?	# of days
12	Recurrent episode	Did you experience any episodes of depression/anxiety within the last year?	(a) I experienced no episodes (b) I had one episode (c) I had several episodes (d) My symptoms of depression do not occur in episodes
13	Overall success of treatment	Has the treatment of your depression/anxiety over the last year been successful?	(a) Very much (b) Moderately (c) Somewhat (d) Not at all

## Results

### Scope

Given the aim to recommend standard assessments for depression and anxiety, we first defined the target disorders. The group decided to not limit the recommendations to a single disorder but to consider the following spectrum of diseases: Major Depressive Disorder, Depressive Disorder—Not Otherwise Specified, Adjustment Disorder/Depressive Adaptive Disorder, Dysthymia, General Anxiety Disorder, Social Anxiety Disorder, Agoraphobia, Panic Disorder, Post-Traumatic-Stress Disorder, and Obsessive Compulsive Disorder. The aim was that the suggested outcome variables should be responsive to therapy effects from established interventions. Recommendations were limited to adults including adolescents above the age of 14 years as there was agreement across working group members that onset of depression in younger people often occurs before the age of 18 years. Evidence suggests good validity for common adult measures for adolescents (see Online Appendix 3) [27].

### Outcome domains

Following the ICHOM framework [24], the Working Group agreed on four general treatment outcomes: (a) symptom burden, (b) functioning, (c) disease progression and treatment sustainability, and (d) potential side effects of treatments (Tables 2, 3, Online Appendix 4, 6, reference guide at www.ichom.org).

**Table 2:** General outcome measures in the depression and anxiety standard set

Domain	Measure		# of Items	# of translations	Scale	Reliable change index^d^			Cut-Off-Score^i^	Range of score (lowest to highest)	Year published
	Name	Abbreviation				Initial M (SD)^e^	Internal consistency^g,e^	Reliable change of Instrument score^h^			
Symptom burden	Patient Health Questionnaire-9^a,^*	PHQ-9	9	79	Frequency	17.1 (6.1)	0.89	>5	>9	0–27	1999
	Generalized anxiety disorder 7-item scale^b,^*	GAD-7	7	71	Frequency	14.4 (4.7)	0.92	>3	>7	0–21	2006
Functioning	The World Health Organization Disability Assessment Schedule 2.0 12-Item Version^c^	WHODAS 2.0 12-Item	12	13	Intensity	27.14 (17.1)^f^	0.96^f^	>9^f^	n/a	0–100^j^	2010

*M* mean, *SD* standard deviation, *sqrt* square root

* Score has to be converted to common metric *T*-Score

^a^see Reference [62]

^b^see Reference [30]

^c^see Reference [36]

^d^To calculate the RCIs, reliability indices, sample means, and score distributions were taken from the original validation studies. Patients’ mental health status should be classified based on the RCIs and cut-off-scores. Cut-off scores determine whether patients are likely to meet diagnostic criteria for an existing mental health disorder. If the difference of instrument scores (*T*2−*T*1 = Δ) is more negative than -RCI (negative Δ < −RCI), the patient is classified as “deteriorated”. If Δ < ±RCI, irrespective of the cut-off, the patient is classified as “unchanged”. If Δ > RCI and the cut-off is not achieved, the patient is classified as “improved”. Finally, if Δ > RCI and the instrument cut-off is achieved, the patient is classified as “recovered” [38]

^e^Information taken from original validation studies (see a-c)

^f^As data from 12-item version were not available, information was taken from validation study for 36 item version in depressed patients [63]

^g^Cronbach’s α

^h^Reliable Change index (RCI) calculated from Cronbach’s α and initial SD (patients with positive diagnosis) from original validation studies (see a-c); formula used for criterion level, based on change that would happen less than 5% of the time by unreliability of measurement alone: RCI = 1.96 × SD × sqrt (2) × sqrt (1 − *α*); results were rounded to integers if necessary for interpretation of the scale

^i^Instrument score that allows to make a diagnosis (confidence interval depends on measure)

^j^0–4 Item Scale; 0 = None; 4 = Extreme or cannot do. This summed score is divided by 48 and multiplied by 100 in order to give a final percentage. Functioning level ranges from 0% (full function) to 100% (no function)

**Table 3:** Assessment sets, domains, number of items, and estimated time for completion of the depression and anxiety standard set

	BL (baseline set)	TM (treatment monitoring set)	AA (annual outcome assessment)
Case-mix factors	Age Sex Educational level Living status Work status Social Support Comorbidities Prior episodes of depression/anxiety Duration of symptoms Prior treatment Outcome expectancy	Current treatment	Living status Work status Comorbidities Prior and current treatment Social support Outcome expectancy
Outcomes	Symptom burden (PHQ-9 and GAD-7) Medication side effects Functioning (WHODAS 2.0) Absenteeism	Symptom burden (PHQ-9 and GAD-7) Single Functioning item (PHQ-9/GAD-7 supplement) Medication side effects Time to recovery	Symptom burden (PHQ-9 and GAD-7) Medication side effects Recurrent episode Functioning (WHODAS 2.0) Absenteeism Overall success of treatment Change of mental health status RCI
# of Items	47	19	45
Time [min]*	13	5	12

*WHODAS 2.0* The World Health Organization Disability Assessment Schedule 2.0 12-Item Version, *RCI* reliable change index, *PHQ-9* Patient Health Questionnaire-9

* Information on time to complete surveys varies between 2·5 and 5 items per minute according to source. A mean of 3.75 was employed to calculate durations

Prioritization of treatment outcomes (teleconference #1, see Online Appendix 4) based on Porter’s outcome measures hierarchy [24, 28] resulted in the exclusion of the domains “survival” and “long-term consequences of therapy” as they were felt to be less relevant for depression or anxiety. “Degree of health achieved or maintained,” “Time to recovery,” “disutility of care or treatment process,” and “sustainability of health” were included resulting in 13 final outcomes (i.e., symptoms of depression/anxiety, social functioning, medication side effects, etc.; see Online Appendix 4).

A comprehensive literature review to find potential instruments measuring these outcomes was carried out between July 2nd and 21st, 2014 (Fig. 1). After removing duplicates, instruments for children, instruments in other languages than English, and instruments assessing depression or anxiety as a comorbidity of other disorders (e.g., depression following a stroke), a total of 80 instruments were retained which assess depressive, general or specific anxiety symptoms, or disease-related functioning. These instruments were reduced further based on aforementioned inclusion criteria resulting in 23 instruments (see Fig. 1 and “Methods” section).

#### Symptom burden

Fifteen scales were analyzed in detail based on aforementioned considerations (Fig. 1, Online Appendix 4, 6), discussed within the working group (teleconference #2) and voted on. The depression subscale of the Patient Health Questionnaire [29], the 9-item PHQ-9, was selected to measure depressive symptoms for patients with depressive disorders, and its anxiety subscale Generalized Anxiety Disorder 7-Item scale (GAD-7) [30] was chosen to measure anxiety symptoms in patients with anxiety spectrum disorders. These scales were selected due to their excellent psychometric properties, the large amount of translations available, the availability of population norms, cross-cultural validation for a large number of languages (www.phqscreeners.com), and their acceptance in the scientific community [30].

In making this recommendation, we recognized that the GAD-7 is a generic measure of anxiety developed primarily to assess generalized anxiety disorders (GAD) and may fail to properly measure the impact of treatment on more specific anxiety disorders (e.g., in cases where avoidance reduces the anxious affect as in housebound agoraphobia or in cases with intrusive memories, compulsions or avoidance). For this reason, institutions desiring a more comprehensive assessment of specific anxiety disorders may wish to complement the GAD-7 with additional instruments. In Online Appendix 8 and 9 (online supplements), the instruments used in the IAPT program are listed for reference purposes. We did not include these measures as part of the formal standard set as most remain research instruments and would benefit from additional optimization (e.g. reduction of item burden) before use in clinical practice.

Recent studies and initiatives have emerged using item response theory methods to develop large item banks allowing to score different instruments measuring the same construct on one common—instrument-independent—metric [9, 31, 32]. These item banks provide an opportunity to move away from instrument defined measurements to construct defined measurements; similar to the assessment of biomedical markers, where for example, measurement of an HbA1c level is independent from the manufacture of the laboratory device. There are several efforts in this respect (www.common-metrics.org), the one receiving the most public support today is the development of the Patient-Reported Outcomes Measurement Information System (PROMIS^®^, www.nihpromis.org) [33], cross-funded by all National Institutes of Health the U.S.. Thus, to facilitate comparisons of our current recommendations with other existing instruments and to ensure its forward-compatibility we propose that raw scores of the PHQ-9, GAD-7 should be converted to the common-metric provided by the PROMIS initiative (referred to as “PROMIS metric” throughout the text). This can be easily achieved using look-up tables (Table A2 in [31, 32], also included in the reference guide) or freely available software (www.common-metrics.org).

#### Functioning

Given the large body of evidence suggesting that depression and anxiety disorders are associated with impaired functioning, the Working Group recommended its inclusion in the standard set [34]. However, functioning is a broad domain with often lengthy assessments, which reduces feasibility in clinical practice, particularly in community-based, frontline care settings. To counterbalance these considerations, the Working Group recommended a more comprehensive assessment at baseline and annual follow-up and a shorter one-item measure during treatment. Due to its availability in many languages and general population reference data, we selected the World Health Organization Disability Schedule 2.0 (WHODAS 2.0) 12-item self-rating version to measure physical, social, and occupational functioning (Tables 2, 3, Online Appendix 8, 9) at baseline and at annual follow-ups [35, 36]. For ongoing treatment, a single item from the PHQ-9 (additional item) that assesses the difficulties of daily life functioning patients attribute to their symptoms, which has been found to correlate highly with other longer functioning scales was selected [29, 34].

#### Disease progression and treatment sustainability

Depression and anxiety are remitting and relapsing in nature, prompting the Working Group’s desire to capture the time to recovery and sustainability of recovery over time. We recommend capturing time to recovery using the reliable change index (RCI) on symptom burden assessments that are collected throughout the process of care. The RCI helps determine whether changes in instrument score indicate a clinically meaningful (reliable) alteration of symptoms rather than an artifact of measurement error (Table 2, Online Appendix 8) [37, 38]. To assess sustainability of recovery, in addition to the annual assessment of symptom burden and functioning, we developed a single item regarding patients’ self-report of depressive episodes during the past year (Table 1, #6, and #12). Workplace absenteeism, a primary driver of overall economic costs was also prioritized for inclusion, defined as the number of days missed during the last month due to illness (Table 1, #11). Finally, we prioritized patients’ perceived success of treatment as this appraisal is one of the best indicators for good treatment outcome (Table 1, #9) [39].

#### Treatment side effects

Primarily informed by the experience of patients in the Working Group, treatment side effects were included in the Standard Set. Mild side effects with intake of antidepressants are very common and we recognize that clinicians often accept these side effect profiles, but the awareness of side effects and the improved ability to project which side effects a patient was most likely to experience, was considered of sufficient importance to warrant their inclusion. As no short but well-validated instrument was found for assessing treatment side effects, we developed a simple assessment for proposed use and future validation (Table 1, #10).

### Baseline characteristics

A primary goal of this effort is to ensure comparisons of treatment outcomes across providers. As such, we sought to identify a minimum set of baseline characteristics to allow for future case-mix adjustments. In identifying case-mix factors, the Working Group agreed on the four following areas: demographics, baseline functional status, baseline clinical status, and prior treatments.

#### Demographic factors

Age, sex, socioeconomic status (SES), and living situation were included as key demographic variables and defined in line with other ICHOM Standard Sets (Table 1, #1–4). Patient-reported highest level of education can be collected as a surrogate measurement of SES [40], as patients generally feel comfortable reporting this information and it can be compared across countries using the International Standard Classification of Education [41]. Individual countries may elect to complement education level with additional measures of SES if available, such as income-based or postal-code based measures. Although influence of living situation on outcome has not yet been systematically investigated, clinical experience indicates that it influences treatment effect. We recommend collecting living situation using a simple assessment routinely collected across the National Health Service PROMs program [42].

#### Baseline functional status

We recommend collecting all outcome measures at baseline, including the WHODAS 2.0 to allow for changes in status to be calculated over time. We also recommend collecting work (Table 1, #5) status and social support at baseline as these factors are predictors of treatment success [43]. As with other ICHOM Standard Sets, a single item was used to assess work status. To capture social support, we recommend using four items of the “Medical Outcomes Study—Social Support Survey” (MOS-SSS) [44]. This instrument yielded a stable factor structure even with a reduced number of items and within assessments in low and middle income countries [45].

#### Baseline clinical status

To allow segmentation of patients for analyses, we recommend recording clinical diagnoses using established classification systems (i.e., ICD or DSM). In addition, we recommend capturing mental and general medical comorbidities, as they have been shown to influence treatment outcomes [46]. We recommend using the Self-administered Comorbidity Questionnaire (SCQ) extended by a list of mental comorbidities to capture these factors [47, 48]. Although relatively unknown, the SCQ has shown to predict functional outcome with equivalence to medical record based Charlson Comorbidity Index [49]. Patients’ expectation regarding success of their treatment is also strongly related to treatment outcome and we elected to include an adapted single item from the credibility/expectancy questionnaire: “How successful do you think will the therapy be in reducing your symptoms?” [39]. Adaptation of this questionnaire to a single item in previous studies has shown good applicability [50].

#### Prior treatment and course of disease

Prior treatments and duration of disease have also been shown to influence treatment outcomes [51]. We developed a single item to capture the use of mental health treatments (i.e., medication, psychotherapy, or other) during the last year (Table 1, #8) as well as single items on prior episodes of depression and duration (in months) of the current episode (Table 1, #6–7).

### Assessment time points

Throughout the consensus process, the assessment scheme was revised twice. Initial online voting supported the recommendation of monthly assessments during treatment, assessments on every third month during the first year after completion of treatment, and a two-year follow-up period. Our final recommendation arose from the thinking that beginning and duration of treatments may vary significantly across patients and that completeness of pre-post treatment data could be improved by frequent assessments during active treatment. In addition, some fixed (annual) assessments would allow for better comparability between patient groups.

**Fig. 2:**
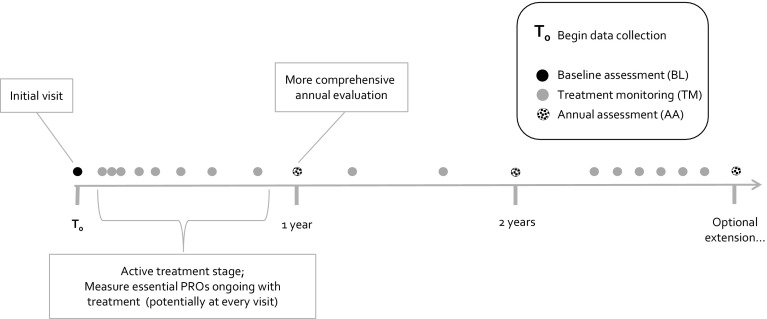
Follow-up timeline for the depression and anxiety standard set. Proposed and optional assessment time points for subsets included in the ICHOM depression and anxiety standard set. *BL* baseline assessment, *TM* treatment monitoring, *AA* annual assessment

Finally, we designed the standardized set with a baseline assessment and two follow-up modules, one focused on capturing changes in symptom burden during active treatment (treatment monitoring (TM)), and a more comprehensive annual outcome assessment (annual assessment (AA)) to allow for research and benchmarking activities with data collected at the same time points. The IAPT program has shown that regular data collection during a course of treatment helps guide therapy and ensures very high (up to 97%) pre-post data completeness [52]. In order to be more helpful in clinical practice, we designed the short TM as a set of variables that are very succinct and focused to inform clinical decision making (Fig. 2). Although it is usually recommended the AA be collected at least annually, we do encourage institutions that wish to conduct more frequent follow-up to do so. As some baseline characteristics may change over the course of the treatment process (living status, work status, comorbidities, prior and current treatment, social support, and outcome expectancy), we also recommend they be updated annually.

### Reference guide

A freely available reference guide that further describes each instrument and provides detailed information about how to calculate scale scores including PROMIS conversion tables and RCIs is accessible online (www.ichom.org).

## Discussion

As health systems around the world shift their attention to measuring the value (outcomes relative to cost) of the services they fund and deliver, there is a need to align on what constitutes the outcomes most relevant to patients. An internationally standardized dataset would satisfy the diverse needs of patients, clinicians, researchers, and policy makers, including: (1) improved communication and decision making between patients and their providers on what treatment plans would best suit their needs, (2) improved monitoring of the impact of treatments across populations of patients, including informing comparative effectiveness studies, and (3) consumer-facing comparisons of the relative outcome performance of different care facilities.

For patients with mental health disorders, such a dataset will mainly rely on the patients’ self-assessment. However, although patient-reported outcomes have been receiving more attention over the last years, the measurement of PROs is still not as established as the measurement of biomedical markers. One reason for this is a lack of standardization. The goal of the present initiative was to recommend a basic set of outcome assessments for depression and anxiety disorders that can align existing and newly developing initiatives and facilitate more global collaboration in the move towards outcome- or value-based systems.

In contrast to other consensus efforts, patient representatives (LL, DS) were full members of the working group; hence, they were involved in the formal development process of the standard set as well as preparation of the manuscript. They participated in every teleconference and the online surveys and had the same voting rights as any other member of the group. For example, inclusion of medication side effects for follow-up assessments was regarded as important by patient representatives and finally included in the standard set. Thus, patient representatives were heavily involved in the decision-making process.

### Limitations

#### Group of experts

Our intention was to include a wide spectrum of patient representatives, clinicians, and researchers from all continents in our group, resulting in a group of 24 members to discuss the different steps of this proposal; however, there are many other well-known scientists, clinicians or patient representatives that could have provided their valuable expertise, and different opinions might have been expressed. By extensive literature reviews, and applying a Delphi technique to document our decision-making process, we strived to achieve a high level of transparency. Nevertheless, like any similar efforts, a different group of participants could have agreed on different recommendations.

#### Outcome measures

The main challenge during the entire project was to propose a set of domains and variables comprehensive enough to be meaningful but short enough to be implementable on a large scale and in a variety of settings. With a focus on feasibility of implementation, we focused on measures considered to be most helpful in the clinical setting. From a scientific perspective, a larger set of domains would be of interest, and we consider this set a foundation upon which other measures might be added for specific research questions.

Another limitation is the inclusion of adhoc items, primarily assessing parts of the patients’ medical history to allow for case-mix adjustment across patient groups. Including these types of items (i.e., to assess prior episodes of the disorder or current work status) was essential for the current value-based approach. Prospectively collected data over the next years will facilitate validation of the proposed standard set, which will deliver crucial information on whether the chosen variables work or whether potential adjustments are necessary.

During the consensus process, there were extensive discussions within the working group as to whether the different depressive disorders such as major depression, dysthymia, or double depression can be assessed with one single tool to measure depressive symptoms (i.e., the PHQ-9), and in particular whether anxiety symptoms from different anxiety spectrum disorders, like General Anxiety Disorder, Social Anxiety Disorder, Agoraphobia, Panic Disorder, Post-Traumatic-Stress Disorder, or Obsessive Compulsive Disorder, can be assessed with another single tool either (i.e., the GAD-7). To prioritize simplicity and standardization we recommended, nevertheless, this as the rather parsimonious approach. The measures proposed to assess symptom burden, i.e., the PHQ-9 and GAD-7, have been widely used to assess different disease states and manifestations of depression and anxiety, respectively [53]. However, we are aware that in particular for patients with phobic disorders additional questionnaires may be required to appropriately document the symptom burden of the individual patient (Online Appendix 8 and 9; online supplements).

Another important consideration was the accessibility of the tools worldwide. This criterion excluded many alternative tools. However, the suggestion to report the raw scores of the PHQ-9 and GAD-7 on a common metric based on modern IRT-methods should allow continued adoption of future instruments that are compatible with such an approach. Among several methods to make scores from different instruments comparable which have been described in the literature [54, 55], we decided to recommend an IRT-approach as this promises to provide an instrument-independent metric for many tools at one time [9], and not just a method to compare one score to another, like an regression approach. Among the few IRT-based metrics which are available today, we decided to use the PROMIS metric, as it received the most public support over the last decade, and is in our opinion the most likely to be widely accepted in the future [31, 32]. However, we are aware that in particular for the more heterogeneous anxiety construct there are still several scientific issues which are currently discussed from those applying these methods [56].

#### Stakeholders

We recognized from the beginning that a single standardized set cannot meet the expectations of all potential stakeholders. Our focus was first on meeting the needs of practicing clinicians to better communicate with their patients and assess the impact of their care. Other stakeholders, such as administrators and economists, may have preferred metrics that are used across diseases for utility measurement (e.g., EQ-5D) [57]. For clinicians, utility-based instruments are insufficiently sensitive to change and unrelated to the disease construct, making interpretability and actionability in the clinical context more difficult. Certainly, research programs wishing to compare utilities alongside disease-focused impact would be welcome to add such measures to their battery of assessments.

#### Evaluation

Measures and items included in the standard set have been carefully chosen with regard to their psychometric properties and availability of validation studies. Some new items had to be adapted or developed to allow for case-mix adjustment (Table 1), evaluation of these items is pending. In addition, as the standard set had just been translated into other languages, future cross-cultural validation studies of the entire set are warranted. For the main outcome instruments such as PHQ-9, GAD-7, and WHODAS 2.0 available evidence has already shown cross-cultural validity [58–61].

#### Implementation

The Working Group recognized that many implementation challenges remain to achieve the anticipated impact of this set. A number of pilot institutions, including selected members of this Working Group, are currently implementing the set with the intention of sharing their experience on the cost and quality impact. In many health systems, the collection of outcomes data is becoming more routine through the use of health information technology, which should facilitate the adoption of these recommendations. Moreover, in health systems with low adoption of such technologies, paper and pencil still provides a cheap and effective mode of data capture. The recommendation of license-free measures further supports adoption.

## Conclusion

Through the efforts reported in this paper, we defined a parsimonious set of patient-reported outcome measures recommended to be applied in patients with depression and anxiety disorders. We hope this can become an important step towards improving the quality and value of care for persons living with depression and anxiety around the world.

## Electronic supplementary material

Below is the link to the electronic supplementary material.

Supplementary material 1 (PDF 764 kb)

Supplementary material 2 (PDF 495 kb)

Supplementary material 3 (PDF 495 kb)

Supplementary material 4 (PDF 446 kb)
