# Supplementary material for: Standardization of health outcomes assessment for depression and anxiety: recommendations from the ICHOM Depression and Anxiety Working Group
Source: Qual Life Res. 2017 Aug 7;26(12):3211–25. doi: 10.1007/s11136-017-1659-5 (PMC5681977; doi:10.1007/s11136-017-1659-5)
Supplement: Supplementary file 1 — Supplementary material 1 (PDF 764 kb) [file 11136_2017_1659_MOESM1_ESM.pdf]

Standardization of health outcome assessment for depression and anxiety: recommendations from the ICHOM Depression and Anxiety Working Group - Appendix

Authors:

Alexander Obbarius*; Lisa van Maasakkers; Lee Baer; David M. Clark; Anne G. Crocker; Edwin de Beurs; Paul M. G. Emmelkamp; Toshi A. Furukawa; Erik Hedman; Maria Kangas; Lucie Langford; Alain Lesage; Doris M. Mwesigire; Sandra Nolte; Vikram Patel; Paul A. Pilkonis; Harold A. Pincus; Roberta A. Reis; Graciela Rojas; Cathy Sherbourne; Dave Smithson; Caleb Stowell; Kelly Woolaway-Bickel; Matthias Rose.

***Corresponding author:** Alexander Obbarius, MD

Postal Address: Department of Psychosomatic Medicine
Charité – Universitätsmedizin Berlin
Hindenburgdamm 30
12203 Berlin
Germany

Telephone: +4930450653890

Fax: +4930450553900

Email: alexander.obbarius@charite.de

Appendix 1: Preliminary list (n = 80) of instruments considered for the D+A Standard Set		
**Instrument**	**Full Name**	**Reference**
Depression (n = 29)		
BDI	Beck Depression Inventory	Beck, A. T., Ward, C. H., Mendelson, M., Mock, J., & Erbaugh, J. (1961). An inventory for measuring depression. *Arch Gen Psychiatry, 4*, 561-571.
BDI - FS	Beck Depression Inventory-FastScreen	Beck, A. T., Steer, R. A., & Brown, G. K. (2000). *Manual for the Beck Depression Inventory-Fast Screen for medical patients*. San Antonio, TX: Psychological Corporation.
BDI-II	Beck Depression Inventory - II	Beck, A. T., Steer, R. A., & Brown, G. K. (1996). *Manual for the Beck Depression Inventory-II*. San Antonio, TX: Psychological Corporation.
BSI Depression	Brief Symptom Inventory - Depression	Derogatis, L. R., & Melisaratos, N. (1983). The Brief Symptom Inventory: an introductory report. *Psychol Med, 13*(3), 595-605.
CDS	Carroll Rating Scale for Depression	Carroll, B. J., Feinberg, M., Smouse, P. E., Rawson, S. G., & Greden, J. F. (1981). The Carroll rating scale for depression. I. Development, reliability and validation. *Br J Psychiatry, 138*, 194-200.
CES-D	Center for Epidemiologic Studies Depression Scale	Radloff, L. S. (1977). The CES-D Scale: A self-report depression scale for research in the general population. *Applied Psychological Measurement, 1*, 385-401, doi:10.1177/014662167700100306.
CMDQ	Common Mental Disorder Questionnaire	Christensen, K. S., Fink, P., Toft, T., Frostholm, L., Ornbol, E., & Olesen, F. (2005). A brief case-finding questionnaire for common mental disorders: the CMDQ. *Fam Pract, 22*(4), 448-457, doi:10.1093/fampra/cmi025.
DASS	Depression Anxiety Stress Scales	Lovibond, S. H., & Lovibond, P. F. (1995). *Manual for the Depression Anxiety Stress Scales* (2nd ed.). Sydney: Psychological Foundation.
DASS-21	Depression Anxiety Stress Scales Short Form	Henry, J. D., & Crawford, J. R. (2005). The short-form version of the Depression Anxiety Stress Scales (DASS-21): construct validity and normative data in a large non-clinical sample. *Br J Clin Psychol, 44*(Pt 2), 227-239, doi:10.1348/014466505X29657.
DUKE-AD	Duke Anxiety - Depression Scale	Parkerson, G. R., Jr., Broadhead, W. E., & Tse, C. K. (1990). The Duke Health Profile. A 17-item measure of health and dysfunction. *Med Care, 28*(11), 1056-1072.
GRID-HAMD	GRID Hamilton Depression Rating Scale	Williams, J. B., Kobak, K. A., Bech, P., Engelhardt, N., Evans, K., Lipsitz, J., et al. (2008). The GRID-HAMD: standardization of the Hamilton Depression Rating Scale. *Int Clin Psychopharmacol, 23*(3), 120-129, doi:10.1097/YIC.0b013e3282f948f5.
HADS	Hospital Anxiety and Depression Scale	Zigmond, A. S., & Snaith, R. P. (1983). The hospital anxiety and depression scale. *Acta Psychiatr Scand, 67*(6), 361-370.
HAM-D	Hamilton Rating Scale for Depression	Hamilton, M. (1960). A rating scale for depression. *J Neurol Neurosurg Psychiatry, 23*, 56-62.
HANDS	Harvard Department of Psychiatry/NDSD scale	Baer, L., Jacobs, D. G., Meszler-Reizes, J., Blais, M., Fava, M., Kessler, R., et al. (2000). Development of a brief screening instrument: the HANDS. *Psychother Psychosom, 69*(1), 35-41, doi:12364.
IDS	Inventory of Depressive Symptomatology	Rush, A. J., Gullion, C. M., Basco, M. R., Jarrett, R. B., & Trivedi, M. H. (1996). The Inventory of Depressive Symptomatology (IDS): psychometric properties. *Psychol Med, 26*(3), 477-486.
ISR Depression	ICD-10-Symptom-Rating - Depression	Tritt, K., von Heymann, F., Zaudig, M., Zacharias, I., Sollner, W., & Loew, T. (2008). [Development of the "ICD-10-Symptom-Rating"(ISR) questionnaire]. *Z Psychosom Med Psychother, 54*(4), 409-418, doi:10.13109/zptm.2008.54.4.409.
MACL	Mood Adjective Checklist	Nowlis, V. (1966). *Development of a Mood Adjective Check List (macl)*. Rochester, NY: Defense Technical Information Center.
MADRS-S	Montgomery Asberg Depression Rating Scale	Montgomery, S. A., & Asberg, M. (1979). A new depression scale designed to be sensitive to change. *Br J Psychiatry, 134*, 382-389.

MDI	Major Depression Inventory	Bech, P., Rasmussen, N. A., Olsen, L. R., Noerholm, V., & Abildgaard, W. (2001). The sensitivity and specificity of the Major Depression Inventory, using the Present State Examination as the index of diagnostic validity. *J Affect Disord, 66*(2-3), 159-164.
MHI-5	Mental Health Index	Berwick, D. M., Murphy, J. M., Goldman, P. A., Ware, J. E., Jr., Barsky, A. J., & Weinstein, M. C. (1991). Performance of a five-item mental health screening test. *Med Care, 29*(2), 169-176.
PHQ-2	Patient Health Questionnaire - 2 Items	Lowe, B., Kroenke, K., & Grafe, K. (2005). Detecting and monitoring depression with a two-item questionnaire (PHQ-2). *J Psychosom Res, 58*(2), 163-171, doi:10.1016/j.jpsychores.2004.09.006.
PHQ-9	Patient Health Questionnaire - 9 Items	Kroenke, K., Spitzer, R. L., & Williams, J. B. (2001). The PHQ-9: validity of a brief depression severity measure. *J Gen Intern Med, 16*(9), 606-613.
PROMIS-Short Form 4	Patient-Reported Outcomes Measurement Information System - Depression Short Form 4 Items	Choi, S. W., Reise, S. P., Pilkonis, P. A., Hays, R. D., & Cella, D. (2010). Efficiency of static and computer adaptive short forms compared to full-length measures of depressive symptoms. *Qual Life Res, 19*(1), 125-136, doi:10.1007/s11136-009-9560-5.
PROMIS-Short Form 6	Patient-Reported Outcomes Measurement Information System - Depression Short Form 6 Items	
PROMIS-Short Form 8	Patient-Reported Outcomes Measurement Information System - Depression Short Form 8 Items	
QIDS-SR	Quick Inventory of Depressive Symptomatology - self report	Rush, A. J., Trivedi, M. H., Ibrahim, H. M., Carmody, T. J., Arnow, B., Klein, D. N., et al. (2003). The 16-Item Quick Inventory of Depressive Symptomatology (QIDS), clinician rating (QIDS-C), and self-report (QIDS-SR): a psychometric evaluation in patients with chronic major depression. *Biol Psychiatry, 54*(5), 573-583.
RDS	Rand 8-item Depression Screener	Burnam, M. A., Wells, K. B., Leake, B., & Landsverk, J. (1988). Development of a brief screening instrument for detecting depressive disorders. *Med Care, 26*(8), 775-789.
SDS	Zung Self-Rating Depression Scale	Zung, W. W. (1965). A Self-Rating Depression Scale. *Arch Gen Psychiatry, 12*, 63-70.
WHO-5	WHO-Five Well-being Index	Bech, P., Gudex, C., & Johansen, K. S. (1996). The WHO (Ten) Well-Being Index: validation in diabetes. *Psychother Psychosom, 65*(4), 183-190.
Anxiety (n = 32)		
ACQ	Agoraphobic Cognitions Questionnaire	Chambless, D. L., Caputo, G. C., Bright, P., & Gallagher, R. (1984). Assessment of fear of fear in agoraphobics: the body sensations questionnaire and the agoraphobic cognitions questionnaire. *J Consult Clin Psychol, 52*(6), 1090-1097.
ADDQ	Anxiety Disorder Diagnostic Questionnaire	Norton, P. J., & Robinson, C. M. (2010). Development and evaluation of the anxiety disorder diagnostic questionnaire. *Cogn Behav Ther, 39*(2), 137-149, doi:10.1080/16506070903140430.
ASQ-15	Anxiety Screening Questionnaire	Wittchen, H. U., & Boyer, P. (1998). Screening for anxiety disorders. Sensitivity and specificity of the Anxiety Screening Questionnaire (ASQ-15). *Br J Psychiatry Suppl*(34), 10-17.
BAI	Beck Anxiety Inventory	Beck, A. T., Epstein, N., Brown, G., & Steer, R. A. (1988). An inventory for measuring clinical anxiety: psychometric properties. *J Consult Clin Psychol, 56*(6), 893-897.
FQ	Fear Questionnaire	Van Zuuren, F. J. (1988). The fear questionnaire. Some data on validity, reliability and layout. *Br J Psychiatry, 153*, 659-662.
GAD-7	General Anxiety Disorder	Spitzer, R. L., Kroenke, K., Williams, J. B., & Lowe, B. (2006). A brief measure for assessing generalized anxiety disorder: the GAD-7. *Arch Intern Med, 166*(10), 1092-1097, doi:10.1001/archinte.166.10.1092.
Ham-A	Hamilton Anxiety Scale	Hamilton, M. (1959). The assessment of anxiety states by rating. *Br J Med Psychol, 32*(1), 50-55.
IES-R	Impact of Events Scale Revised	Weiss, D. S., & Marmar, C. R. (1997). The Impact of Event Scale-Revised. In J. P. Wilson, & T. M. Keane (Eds.), *Assessing psychological trauma and PTSD* (pp. 399–411). New York: Guilford Press.

LSAS-SR	Liebowitz Social Anxiety Scale - self reported	Liebowitz, M. R. (1987). Social phobia. *Mod Probl Pharmacopsychiatry, 22*, 141-173.
MASQ	Mood and Anxiety Symptoms Questionnaire	Watson, D., Weber, K., Assenheimer, J. S., Clark, L. A., Strauss, M. E., & McCormick, R. A. (1995). Testing a tripartite model: I. Evaluating the convergent and discriminant validity of anxiety and depression symptom scales. *J Abnorm Psychol, 104*(1), 3-14.
MI	Mobility Inventory	Chambless, D. L., Caputo, G. C., Jasin, S. E., Gracely, E. J., & Williams, C. (1985). The Mobility Inventory for Agoraphobia. *Behav Res Ther, 23*(1), 35-44.
Mini-SPIN	Social Phobia Inventory - Abbreviated Version	Connor, K. M., Kobak, K. A., Churchill, L. E., Katzelnick, D., & Davidson, J. R. (2001). Mini-SPIN: A brief screening assessment for generalized social anxiety disorder. *Depress Anxiety, 14*(2), 137-140.
OCI	Obsessive Compulsive Inventory	Foa, E. B., Kozak, M. J., Salkovskis, P. M., Coles, M. E., & Amir, N. (1998). The validation of a new obsessive–compulsive disorder scale: The Obsessive–Compulsive Inventory. *Psychol Assess, 10*(3), 206-214.
PAI	Panic Appraisal Inventory	Feske, U., & de Beurs, E. (1997). The Panic Appraisal Inventory: psychometric properties. *Behav Res Ther, 35*(9), 875-882.
PAS	Panic and Agoraphobia Scale	Bandelow, B. (1995). Assessing the efficacy of treatments for panic disorder and agoraphobia. II. The Panic and Agoraphobia Scale. *Int Clin Psychopharmacol, 10*(2), 73-81.
PCL	PTSD checklist	Blanchard, E. B., Jones-Alexander, J., Buckley, T. C., & Forneris, C. A. (1996). Psychometric properties of the PTSD Checklist (PCL). *Behav Res Ther, 34*(8), 669-673.
PDS	Posttraumatic Diagnostic Scale	Foa, E. B., Cashman, L., Jaycox, L., & Perry, K. (1997). The validation of a self-report measure of posttraumatic stress disorder: The Posttraumatic Diagnostic Scale. *Psychol Assess, 9*, 445–451.
PDSS-SR	Panic Disorder Severity Scale- Self Report	Houck, P. R., Spiegel, D. A., Shear, M. K., & Rucci, P. (2002). Reliability of the self-report version of the panic disorder severity scale. *Depress Anxiety, 15*(4), 183-185, doi:10.1002/da.10049.
PROMIS-Short Form 4	Patient-Reported Outcomes Measurement Information System - Anxiety Short Form 4 Items	Pilkonis, P. A., Choi, S. W., Reise, S. P., Stover, A. M., Riley, W. T., Cella, D., et al. (2011). Item banks for measuring emotional distress from the Patient-Reported Outcomes Measurement Information System (PROMIS(R)): depression, anxiety, and anger. *Assessment, 18*(3), 263-283, doi:10.1177/1073191111411667.
PROMIS-Short Form 8	Patient-Reported Outcomes Measurement Information System - Depression Short Form 8 Items	
PSS-SR	Posttraumatic Stress Disorder Symptom Scale	Falsetti, S. A., Resnick, H. S., Resick, P. A., & Kilpatrick, D. (1993). The Modified PTSD Symptom Scale: A brief self-report measure of posttraumatic stress disorder. *The Behavioral Therapist, 16*, 161-162.
PSWQ	Penn State Worry Questionnaire	Meyer, T. J., Miller, M. L., Metzger, R. L., & Borkovec, T. D. (1990). Development and validation of the Penn State Worry Questionnaire. *Behav Res Ther, 28*(6), 487-495.
SAQ-A30	Social Anxiety Questionnaire	Caballo, V. E., Salazar, I. C., Irurtia, M. J., Arias, B., Hofmann, S. G., & Team, C.-A. R. (2012). The multidimensional nature and multicultural validity of a new measure of social anxiety: the Social Anxiety Questionnaire for Adults. *Behav Ther, 43*(2), 313-328, doi:10.1016/j.beth.2011.07.001.
SHAI	Health Anxiety Inventory	Salkovskis, P. M., Rimes, K. A., Warwick, H. M., & Clark, D. M. (2002). The Health Anxiety Inventory: development and validation of scales for the measurement of health anxiety and hypochondriasis. *Psychol Med, 32*(5), 843-853.
SIAS+SPS	Social Interaction Scale + Social Phobia Scale	Mattick, R. P., & Clarke, J. C. (1998). Development and validation of measures of social phobia scrutiny fear and social interaction anxiety. *Behav Res Ther, 36*(4), 455-470.
SPAI	Social Phobia and Anxiety Inventory	Turner, S. M., Beidel, D. C., Dancu, C. V., & Sganley, M. A. (1989). An empirically derived inventory to measure social fears and anxiety: The Social Phobia and Anxiety Inventory. *Psychological Assessment: A Journal of Consulting and Clinical Psychology, 1*(1), 35-40.

SPAI-18	Social Phobia and Anxiety Inventory short version	de Vente, W., Majdandzic, M., Voncken, M. J., Beidel, D. C., & Bogels, S. M. (2014). The SPAI-18, a brief version of the social phobia and anxiety inventory: reliability and validity in clinically referred and non-referred samples. *J Anxiety Disord, 28*(2), 140-147, doi:10.1016/j.janxdis.2013.05.003.
SPDQ	Social Phobia Diagnostic Questionnaire	Newman, M. G., Kachin, K. E., Zuellig, A. R., Constantino, M. J., & Cashman-McGrath, L. (2003). The social phobia diagnostic questionnaire: preliminary validation of a new self-report diagnostic measure of social phobia. *Psychol Med, 33*(4), 623-635.
SPIN	Social Phobia Inventory	Connor, K. M., Davidson, J. R., Churchill, L. E., Sherwood, A., Foa, E., & Weisler, R. H. (2000). Psychometric properties of the Social Phobia Inventory (SPIN). New self-rating scale. *Br J Psychiatry, 176*, 379-386.
STAI	State-Trait Anxiety Inventory	Spielberger, C. D., Gorsuch, R. L., Lushene, R., Vagg, P. R., & Jacobs, G. A. (1983). *Manual for the State-Trait Anxiety Inventory*. Palo Alto, CA: Consulting Psychologists Press.
Y-BOCS	Yale-Brown Obsessive Compulsive Scale	Goodman, W. K., Price, L. H., Rasmussen, S. A., Mazure, C., Fleischmann, R. L., Hill, C. L., et al. (1989). The Yale-Brown Obsessive Compulsive Scale. I. Development, use, and reliability. *Arch Gen Psychiatry, 46*(11), 1006-1011.
ZungSAS	Zung Self-rating Anxiety Scale	Zung, W. W. (1971). A rating instrument for anxiety disorders. *Psychosomatics, 12*(6), 371-379, doi:10.1016/S0033-3182(71)71479-0.
Functioning (n = 19)		
CORE-OM	The CORE Outcome Measure	Barkham, M., Margison, F., Leach, C., Lucock, M., Mellor-Clark, J., Evans, C., et al. (2001). Service profiling and outcomes benchmarking using the CORE-OM: toward practice-based evidence in the psychological therapies. Clinical Outcomes in Routine Evaluation-Outcome Measures. *J Consult Clin Psychol, 69*(2), 184-196.
CPFQ	Massachusetts General Hospital Cognitive and Physical Functioning Questionnaire	Fava, M., Iosifescu, D. V., Pedrelli, P., & Baer, L. (2009). Reliability and validity of the Massachusetts general hospital cognitive and physical functioning questionnaire. *Psychother Psychosom, 78*(2), 91-97, doi:10.1159/000201934.
FAQ	Functional Activities Questionnaire	Pfeffer, R. I., Kurosaki, T. T., Harrah, C. H., Jr., Chance, J. M., & Filos, S. (1982). Measurement of functional activities in older adults in the community. *J Gerontol, 37*(3), 323-329.
FIM	Functional Independence Measure	Granger, C. V., Hamilton, B. B., Linacre, J. M., Heinemann, A. W., & Wright, B. D. (1993). Performance profiles of the functional independence measure. *Am J Phys Med Rehabil, 72*(2), 84-89.
FSQ	Functional Status Questionnaire	Jette, A. M., Davies, A. R., Cleary, P. D., Calkins, D. R., Rubenstein, L. V., Fink, A., et al. (1986). The Functional Status Questionnaire: reliability and validity when used in primary care. *J Gen Intern Med, 1*(3), 143-149.
GAF	Global Assessment of Functioning	Hall, R. C. (1995). Global assessment of functioning. A modified scale. *Psychosomatics, 36*(3), 267-275, doi:10.1016/S0033-3182(95)71666-8.
GSDS-II	Groningen Social Disabilities Schedule	Wiersma, D., DeJong, A., & Ormel, J. (1988). The Groningen Social Disabilities Schedule: development, relationship with I.C.I.D.H., and psychometric properties. *Int J Rehabil Res, 11*(3), 213-224.
Katz ADL	Katz Index of Independence in Activities of Daily Living	Shelkey, M., & Wallace, M. (1999). Katz Index of Independence in Activities of Daily Living. *J Gerontol Nurs, 25*(3), 8-9.
PROMIS	Physical Functioning	Rose, M., Bjorner, J. B., Becker, J., Fries, J. F., & Ware, J. E. (2008). Evaluation of a preliminary physical function item bank supported the expected advantages of the Patient-Reported Outcomes Measurement Information System (PROMIS). *J Clin Epidemiol, 61*(1), 17-33, doi:10.1016/j.jclinepi.2006.06.025.
PROMIS	Ability to Participate in Social Roles and Activities	Heinemann, A. W., Kisala, P. A., Hahn, E. A., & Tulsky, D. S. (2015). Development and psychometric characteristics of the SCI-QOL Ability to Participate and Satisfaction with Social Roles and Activities item banks and short forms. *J Spinal Cord Med, 38*(3), 397-408, doi:10.1179/2045772315Y.0000000028.
PROMIS	Satisfaction with Social Roles and Activities	
PSMS	Physical Self-Maintenance Scale	Lawton, M. P., & Brody, E. M. (1969). Assessment of older people: self-maintaining and instrumental activities of daily living. *Gerontologist, 9*(3), 179-186.

SAS-SR	Social Adjustment Scale - Self Report	Weissman, M. M., Prusoff, B. A., Thompson, W. D., Harding, P. S., & Myers, J. K. (1978). Social adjustment by self-report in a community sample and in psychiatric outpatients. *J Nerv Ment Dis, 166*(5), 317-326.
SBS	Social Behaviour Schedule	Wykes, T., & Sturt, E. (1986). The measurement of social behaviour in psychiatric patients: an assessment of the reliability and validity of the SBS schedule. *Br J Psychiatry, 148*, 1-11.
SF-36	Short Form Survey	Ware, J. E., Jr., & Sherbourne, C. D. (1992). The MOS 36-item short-form health survey (SF-36). I. Conceptual framework and item selection. *Med Care, 30*(6), 473-483.
SFQ	Social Functioning Questionnaire	Tyrera, P., Mersona, S., Harrison-Reada, P., Lyncha1, S., Birketta, P., & Onyetta, S. (1990). A pilot study of the effects of early intervention on clinical symptoms and social functioning in psychiatric emergencies. *Irish Journal of Psychological Medicine, 7*(02), 132-134.
SFS2	Social Functioning Scale	Birchwood, M., Smith, J., Cochrane, R., Wetton, S., & Copestake, S. (1990). The Social Functioning Scale. The development and validation of a new scale of social adjustment for use in family intervention programmes with schizophrenic patients. *Br J Psychiatry, 157*, 853-859.
SRP	Social Role Performance Schedule	Sturt, E., & Wykes, T. (1987). Assessment schedules for chronic psychiatric patients. *Psychol Med, 17*(2), 485-493.
WRFQ	Work Role Functioning Questionnaire	Amick, B. C., 3rd, Lerner, D., Rogers, W. H., Rooney, T., & Katz, J. N. (2000). A review of health-related work outcome measures and their uses, and recommended measures. *Spine (Phila Pa 1976), 25*(24), 3152-3160.

Appendix 2: Working group teleconference topics and survey response rates

Topic	Date	Title	Online survey Response Rate [%]
“Kick-Off”	17 June 2014	Launch of the Depression and Anxiety Working Group and setting Scope	77
Teleconference 1	1 July 2014	Selecting Outcome Domains	70
Teleconference 2	29 July 2014	Selecting Outcome Definitions	85
Teleconference 3	26 August 2014	Selecting Baseline Characteristics	90
Teleconference 4	16 September 2014	Selecting Baseline Characteristics Measures	85
Teleconference 5	14 October 2014	Finalize the Standard Set / Prepare for launch and publication	100
Teleconference 6	4 November 2014	Transition to Implementation	-
Teleconference 7	3 March 2015	Revision of the Standard Set*	85

*During the review of the compiled Standard Set, the Working Group expressed concerns about the total length of the recommended Standard Set and decided to break the assessment into two modules: one for treatment monitoring and one focusing on annual outcome assessment. This was subsequently voted on and agreed to by the Working Group.

Appendix 3: Voting percentages of modified Delphi method by working group members on scope.

Domain	Subdomain	Vote for inclusion [%]	Conclusion after first vote	Inclusion [Y=Yes; N=No]	Comments
Patient population	Depressive Adaptive / Adjustment Disorder	60	Debate further	Y	Group decided to include it after discussion as outcomes are the same and as there are no clear differences between symptoms and functions.
	Major depressive disorder	100	Include	Y	
	Depressive disorder – Not otherwise specified	81		Y	
	Dysthymia	76		Y	
	General anxiety disorder	100		Y	
	Phobic disorder	100		Y	
	Panic disorder	100		Y	
	Post-traumatic stress disorder	87		Y	
	Obsessive-compulsive disorder	79		Y	
	Schizophrenic disorder	7	Exclude	N	
	Bipolar disorder	29		N	
	Somatoform disorder	13		N	
	Other main diagnoses	14		N	
Age restriction	>18 years	47	Debate further	>14 years	Group decided to include patients above age of 14 as onset of depression may be before 18. Evidence shows good validity for common adult measures for adolescents*.
	>14 years	41			
	No restriction at all	12			

*Allgaier AK, Pietsch K, Fruhe B, Sigl-Glockner J, Schulte-Korne G. Screening for depression in adolescents: validity of the patient health questionnaire in pediatric care. *Depression and anxiety.* 2012;29(10):906-913.

Appendix 4: Voting percentages of modified Delphi method by working group members on outcome domains.

Domain*	Outcome	Votes** (revotes) for inclusion [%]	Conclusion after first vote	Final inclusion [Y=Yes; N=No]	Comments
Survival				N	Group discussion revealed no relevance for Depression / Anxiety
Degree of health achieved or maintained	Symptoms of depression / anxiety	100	Include	Y	
	Social functioning	100	Include	Y	
	Occupational functioning / work status	100	Include	Y	
	Physical functioning / status	71	Include	Y	
Time to recovery and return to normal activities	Time to symptom relief / sufficient functioning	82	Include	Y	
Disutility of the care or treatment process	Medication side-effects	65	Debate further	Y	Prompted by strong preferences by patients in the group as these are felt very important for treatment adherence.
Sustainability of health / recovery	Overall success of treatment	79	Include	Y	
	Reason for failure of treatment	72 (94)	Include	N	Combined with "Overall success of treatment" after first round, excluded in the last round when item burden was reduced due to concern of reliable collection.
	Recurrence of disease	75	Include	Y	
	Type of on-going treatment	72	Include	Y	Felt essential for segmenting patients for analysis
	# of emergency visits	51	Debate further	N	Excluded after group discussion as it is only relevant in small portion of patients with anxiety disorder.
	# of working days missed	75	Include	Y	
	# of days suffering from disease	75 (88)	Include	N	Excluded in the last round after group discussion as felt to be too difficult to measure retrospectively
Long-term consequences of therapy				N	Group discussion revealed no relevance for Depression / Anxiety

*based on Michael Porters outcome hierarchy: Porter ME. What is value in health care? *N Engl J Med.* 2010;363(26):2477-2481

**Domains were included if % of essential votes + ½ % of "nice to have" votes were above 66%, and debated further if it was between 50% and 66%.

Appendix 5: Voting percentages of modified Delphi method by working group members on baseline characteristics.

Domain	Baseline characteristic	Votes (revotes) for inclusion [%]	Conclusion after first vote	Final Inclusion [Y=Yes; N=No]	Comments
Demographics	Age	100	Include	Y	
	Gender	100	Include	Y	
	Race/Ethnicity	72	Include	N	No measure was found to reliably collect this internationally and the item was then excluded in subsequent rounds.
	Socioeconomics (income/ education)	100	Include	Y	
	Housing situation (e.g. assisted living, with spouse, parents, alone)	83	Include	Y	
	Community-based measures	31	Exclude	N	
	Urbanicity (e.g. measured by postal code)	47	Exclude	N	
	Religion/Belief	11	Exclude	N	
	Sexual orientation	0	Exclude	N	
	Marital status	53	Debate Further	N	Combined with “housing situation” to “living situation”
	Retirement status	33	Exclude	N	
	Sick pay status	33	Exclude	N	
	Health insurance that covers treatment	39	Exclude	N	
Baseline functional status	Work status	100	Include	Y	
	Occupational functioning	89	Include	Y	
	Social functioning	89	Include	Y	
	Physical functioning	89	Include	Y	
	Social support/network	72	Include	Y	
	Duration of sick leave before treatment	28	Exclude	N	
	(inherent) disability	24	Exclude	N	
	Morbidity state	12	Exclude	N	
	Physical activity / sports	17	Exclude	N	
	Maximum walking distance	6	Exclude	N	
Baseline clinical status	Symptoms of depression	95	Include	Y	
	Symptoms of anxiety	95	Include	Y	
	BMI	68 (94)	Include	N	Excluded in the last round when item burden was reduced due to concern of reliable collection.
	Comorbidities	100	Include	Y	
	Chronic disease	63	Debate Further	N	Excluded after group discussion since operationalization was felt to be too difficult.
	Smoking	44	Exclude	N	
	Patients’ motivation and expectancy	78	Include	Y	
	Major life events	53	Debate Further	N	Excluded after group discussion since operationalization was felt to be too difficult.
	Personality factors	35	Exclude	N	
	Level of pain	50	Debate further	N	Excluded after group discussion since it is only relevant in certain comorbidities (such as chronic pain disorder or cancer).

	Positive family history	56	Debate Further	N	Excluded after group discussion as it is not an indicator for treatment success but for individual risk to develop a mental disorder.
	Patients' belief about returning to work	0	Exclude	N	
	Prior episodes	(82)		Y	Added in next round after Working group discussion
Prior Treatment	Prior psychotherapy	95	Include	Y	
	Duration of symptoms	95	Include	Y	
	Medication (prior and current)	100	Include	Y	
	Previous treatment in other psychiatric conditions	47	Exclude	N	

Appendix 6: Voting percentages of modified Delphi method by working group members on outcome measures.

**Outcome**	**Instrument**	**Specifications**							**Conclusion after working group meeting**	**Vote for inclusion [%]**	**Inclusion [Y=Yes; N=No]**	**Comments**
		Year published	Symptom coverage*	Scale	# of items	# of translations	License fee [Y=Yes; N=No]	# of Pubmed hits***				
**Depressive Symptoms**	CES-D	1977	complete	frequency	20	>40	N	2426	Exclude	**6** (pooled response option "other measure for depression")	N	
	WHO-5	1998	incomplete	frequency	5	31	N	187	Exclude		N	
	BDI-II	1996	complete	Y/N	21	17	Y	804	Exclude		N	
	HADS	1983	Incomplete	intensity	7	111	Y	2426	Exclude		N	
	QIDS-SR	2003	Complete	frequency/intensity	16	31	N	86	Exclude		N	
	MADRS-S	1994	complete	intensity	9	42	N	31	Exclude		N	
	SDS	1965	complete	frequency	20	>10	N	569	Exclude		N	
	**PHQ-9****	**1999**	**complete**	**frequency**	**10**	**79**	**N**	**875**	**Include in survey**	**75**	**Y**	
	PROMIS Depression**	2009	incomplete	intensity	8	12	N	55	Include in survey	**19**	N	
**General anxiety disorder**	STAI	1983	complete	frequency/intensity	40	48	Y	1783	Exclude	**0** (pooled response option "other measure for anxiety")	N	
	BAI	1993	complete	intensity	21	13	Y	353	Exclude		N	
	HADS	1983	complete	intensity	7	111	Y	2428	Exclude		N	
	ZungSAS	1971	complete	frequency	20	>10	N	329	Exclude		N	
	PROMIS Anxiety**	2011	complete	intensity	8	6	N	42	Include in survey	25	N	
	**GAD-7****	**2006**	**incomplete**	**frequency**	**7**	**71**	**N**	**126**	**Include in survey**	**75**	**Y**	
Social Phobia	SPIN	2000	complete	intensity	17		N	48	Include in survey	**92**	Y	
	SAQ-A30	2012	complete	intensity	30	8	N	4	Exclude	**8**	N	
	SIAS+SPS	1998	complete	intensity	20+20	4	N	30	Exclude		N	
Agoraphobia	MI	1995	complete	frequency	27	4	N	8	Include in survey	**92**	Y	
	PAS	1995	complete	frequency/intensity	13	>10	Y	32	Exclude	**8**	N	
Post-traumatic symptom disorder	IES-R	1997	complete	intensity	22	>10	N	231	Include in survey	**75**	Y	

	PCL	1993	complete	intensity	17	>10	N	254	Exclude	**25**	N	
Panic Disorder	PDSS-SR	1997	complete	frequency/intensity	7	9	N	62	Include in survey	**92**	Y	
	PAI	1987	complete	intensity	45	1	N	8	Exclude	**8**	N	
Obsessive-compulsive disorder	OCI	1998	complete	intensity	42	>10	N	45	Include in survey	**92**	Y	
	PI-R	1996	complete	intensity	39	<10	N	69	Exclude	**8**	N	
**Functioning (physical, occupational, social)**	WHODAS 2.0 36 Items	2010	complete	intensity	36	13	N	140	Include in survey	**14**	N	
	**WHODAS 2.0 12 Items**	**2010**	**complete**	**intensity**	**12**	**13**	**N**	**140**	**Include in survey**	**69**	**Y**	
	WSAS	2002	complete	intensity	5	n/a	N	88	Include in survey	**14**	N	
	SF36	1989	complete	frequency/intensity	36	>100	Y	>10000	Exclude			
	CORE-OM	2001	incomplete	frequency	34/10	10	N	59	Exclude			
	HAQ	1982	Incomplete	intensity	21	>20	N	1456	Exclude			
	SAS-SR	1976	Incomplete	intensity	54	20	Y	135	Exclude			
	SASS	1997	complete	intensity	21	>10	N	35	Exclude			
	PROMIS	2006 / 2010	complete	intensity	8+8	13/5	N	75/4	Include in survey	**56**	N	
**Time to recovery**	**Reliable Change Index**								**Include in combination with instrument cut-off points**	**75**	**Y**	**See Table 1 and Appendix 7 for more information**

**Outcome**	**Items**	**Conclusion after working group meeting**	**Vote for inclusion [%]**	**Inclusion [Y=Yes; N=No]**	**Comments**
Medication side-effects	Weight gain		**100**	Y	
	Sexual dysfunction		**88**	Y	
	Sleep disturbances		**88**	Y	
	Dry mouth		**50**	Y	Included after group discussion as it is a typical side-effect in classical antidepressant drugs.
	Drowsiness / sedation		**94**	Y	
	Cardiovascular side-effects		**60**	Y	Included after group discussion as it is a

					typical side-effect in psychopharmacological drugs
	Gastrointestinal side-effects		**94**	Y	
Overall success / reasons for failure	Access to treatment		**100**	Y	Group finally decided to integrate items into an “overall success” question to reduce item burden (82% agreement)
	Costs of treatment		**100**		
	Relocation		**92**		
	Worsening of mood symptoms		**88**		
	Interpersonal therapeutic alliance		**100**		
	Poor, unproductive, or hostile therapy sessions		**69**		
	Therapy adherence		**88**		
	Medication side-effects		**100**		
	Treatment ineffectiveness / lack of improvement		**94**		
Recurrence of disease	Physician reported date of clinical diagnosis of recurrence		**83**		Was first suggested as clinician reported. On revision changed to Patient reported outcome (82% agreement)

Full questionnaires and items are shown in the reference guide (www.ichom.org)
*ICD/DSM
**representative samples available
***as an indicator for prominence and dissemination

Appendix 7: Voting percentages of modified Delphi method by working group members on baseline characteristic measures.

**Baseline characteristic**	**Instrument / Item**	**Specifications**					**Conclusion after working group meeting**	**Vote for inclusion [%]**	**Inclusion [Y=Yes; N=No]**	**Comments**
		Year published	# of items	# of translations	License fee [Y=Yes; N=No]	# of Pubmed hits***				
Age	Date of birth		1				Include	**94**	Y	
Gender	Sex at birth		1				Include	**94**	Y	
Socioeconomics (Education)	The level of schooling is defined in each country as per ISCED		1				Include	**100**	Y	
Living Status	living arrangements		1				Include	**89**	Y	
Work status	Current work status		1				Include	**94**	Y	
Social support/network	Medical Outcomes Study Social Support Survey (MOS-SSS)	2014	4	n/a	N	1	Include	**83**	Y	
Comorbidities	Self-Report Charlson Comorbidity Index & mental comorbidities	2003	1	n/a	N	39	Include	**88**	Y	
Patients' motivation and expectancy	Adapted credibility/expectancy questionnaire	2000	1	n/a	N	8	Include	**83**	Y	
Prior episodes	Similar episodes before		1				Include	**82**	Y	Only in patients with depression
Prior psychotherapy	Psychotherapy during last year		1				Include	**94**	Y	
Duration of symptoms	# of month symptoms have been experienced		1				Debate further	**53**	Y	Included after group discussion as duration of an episode/disorder impacts outcome. Item revised in last round (88% agreement).
Medication (prior and current)	Medication intake during last year		1				Include	**100**	Y	

Appendix 8: Optional Measures for specific anxiety disorders as proposed by IAPT

Main Diagnosis	Measure: Name		Measure: Abbreviation	# of Items	# of translations	Scale	Reliable Change Index[6]: Initial M (SD)[7]	Reliable Change Index[6]: Internal consistency[8, 7]	Reliable Change Index[6]: Reliable change of Instrument score[9]	Cut-Off-Score[10]	Range of Score (lowest to highest)	Year published
Social Anxiety Disorder	Social Phobia Inventory[1]		SPIN	17	8	Intensity	41·1 (10·2)	0·87 to 0·94	>9	>18	0 to 68	2000
Agoraphobia	Agoraphobia Mobility Inventory[2]	Avoidance Alone Subscale	MI - AAL	27	>10	Frequency	3·30 (0·99)	0·94	≥0·7	Above an item average of 2.3	1 to 5	1985
		Avoidance Accompanied Subscale	MI - AAC				2·41 (0·70)	0·91	≥0·6	Not relevant for diagnosis of Agoraphobia	1 to 5	
Post-Traumatic-Stress Disorder	Impacts of Events Scale Revised[3]		IES-R	22	>10	Intensity	58·08 (15·18)	0·96	>8	>32	0 to 88	1997
Panic Disorder	Panic Disorder Severity Scale – Self-Report[4]		PDSS-SR	7	9	Frequency and Intensity	9·0 (6·6)	0·92	>5	>7	0 to 28	2002
Obsessive Compulsive Disorder	Obsessive Compulsive Inventory[5]		OCI	42	>10	Intensity	66·33 (31·9)	0·92	>25	>39	0 to 168	1998

M=mean, SD=standard deviation, sqrt = square root

1 Connor KM, Davidson JR, Churchill LE, Sherwood A, Foa E, Weisler RH. Psychometric properties of the Social Phobia Inventory (SPIN). New self-rating scale. The British journal of psychiatry: the journal of mental science. 2000;176:379-386.

2 Chambless DL, Caputo GC, Jasin SE, Gracely EJ, Williams C. The Mobility Inventory for Agoraphobia. Behaviour research and therapy. 1985;23(1):35-44.

3 Creamer M, Bell R, Failla S. Psychometric properties of the Impact of Event Scale - Revised. *Behaviour research and therapy.* 2003;41(12):1489-1496.

4 Houck PR, Spiegel DA, Shear MK, Rucci P. Reliability of the self-report version of the panic disorder severity scale. Depression and anxiety. 2002;15(4):183-185.

5 Foa EB, Kozak MJ, Salkovskis PM, Coles ME, Amir N. The validation of a new obsessive–compulsive disorder scale: The Obsessive–Compulsive Inventory. Psychological assessment. 1998;10(3):206-214.

6 Jacobson NS, Truax P. Clinical significance: a statistical approach to defining meaningful change in psychotherapy research. *J Consult Clin Psychol.* 1991;59(1):12-19. To calculate the RCIs, reliability indices, sample means and score distributions were taken from the original validation studies. Patients' mental health status should be classified based on the RCIs and cut-off-scores (see Table 1). Cut-off scores determine whether patients are likely to meet diagnostic criteria for an existing mental health disorder. If the difference of instrument scores (T2-T1=Δ) is more negative than -RCI (negative Δ < -RCI), the patient is classified as "deteriorated". If Δ < ±RCI, irrespective of the cut-off, the patient is classified as "unchanged". If Δ > RCI and the cut-off is not achieved, the patient is classified as "improved". Finally, if Δ > RCI and the instrument cut-off is achieved, the patient is classified as "recovered".

7 Information taken from original validation studies (see 1-6)

8 Cronbach's α

9 Reliable Change index (RCI) calculated from Cronbach's α and initial SD (patients with positive diagnosis) from original validation studies (see 1-6); formula used for criterion level, based on change that would happen less than 5% of the time by unreliability of measurement alone: RCI=1,96*SD*sqrt(2)*sqrt(1-α); results were rounded to integers if necessary for interpretation of the scale

10 Instrument Score that allows to make a diagnosis (confidence interval depends on measure)

Appendix 9: Overall number of Items and estimated time for completion if measures for specific anxiety disorders are added.										
	**Specific Anxiety Disorders**									
	**Social Anxiety Disorder**		**Agoraphobia**		**Post-Traumatic-Stress Disorder**		**Panic Disorder**		**Obsessive Compulsive Disorder**	
	# of Items	*Time [min]*	*# of Items*	*Time [min]*	*# of Items*	*Time [min]*	*# of Items*	*Time [min]*	*# of Items*	*Time [min]*
BL (baseline set)	57	15	67	18	62	17	47	13	82	22
TM (treatment monitoring set)	29	8	39	11	34	10	19	6	54	15
AO (annual outcome set)	54	15	64	17	59	16	44	12	79	21
*Information on time to complete surveys varies between 2·5 to 5 items per minute according to source. A mean of 3·75 was employed to calculate durations.										
