# Supplementary material for: Standardization of health outcomes assessment for depression and anxiety: recommendations from the ICHOM Depression and Anxiety Working Group
Source: Qual Life Res. 2017 Aug 7;26(12):3211–25. doi: 10.1007/s11136-017-1659-5 (PMC5681977; doi:10.1007/s11136-017-1659-5)
Supplement: Supplementary file 2 — Supplementary material 2 (PDF 495 kb) [file 11136_2017_1659_MOESM2_ESM.pdf]

# ICHOM Annual Assessment for Depression and Anxiety v1

The following questions will assess your current health status helping your health care provider to monitor the treatment success and to acknowledge potential health risk factors.

| | | Much better | A Little Better | About the same | A little Worse | Much worse |
|---|---|---|---|---|---|---|
| 1 | Compared with how you were doing one year ago, would you say that now you are …? | ☐ | ☐ | ☐ | ☐ | ☐ |

| WHODAS 2.0 | In the past 30 days, how much difficulty did you have in: | None | Mild | Moderate | Severe | Extreme or cannot do |
|---|---|---|---|---|---|---|
| 1 | Standing for long periods such as 30 minutes? | ☐ | ☐ | ☐ | ☐ | ☐ |
| 2 | Taking care of your household responsibilities? | ☐ | ☐ | ☐ | ☐ | ☐ |
| 3 | Learning a new task, for example, learning how to get to a new place? | ☐ | ☐ | ☐ | ☐ | ☐ |
| 4 | How much of a problem did you have joining in community activities (for example, festivities, religious or other activities) in the same way as anyone else can? | ☐ | ☐ | ☐ | ☐ | ☐ |
| 5 | How much have you been emotionally affected by your health problems? | ☐ | ☐ | ☐ | ☐ | ☐ |
| 6 | Concentrating on doing something for ten minutes? | ☐ | ☐ | ☐ | ☐ | ☐ |
| 7 | Walking a long distance such as a kilometer [or equivalent]? | ☐ | ☐ | ☐ | ☐ | ☐ |
| 8 | Washing your whole body? | ☐ | ☐ | ☐ | ☐ | ☐ |
| 9 | Getting dressed? | ☐ | ☐ | ☐ | ☐ | ☐ |
| 10 | Dealing with people you do not know? | ☐ | ☐ | ☐ | ☐ | ☐ |
| 11 | Maintaining a friendship? | ☐ | ☐ | ☐ | ☐ | ☐ |
| 12 | Your day-to-day work? | ☐ | ☐ | ☐ | ☐ | ☐ |

| PHQ-9 | Over the last 2 weeks how often have you been bothered by any of the following problems? | Not at all | Several days | More than half the days | Nearly every day |
|---|---|---|---|---|---|
| 1 | Little interest or pleasure doing thing | ☐ | ☐ | ☐ | ☐ |
| 2 | Feeling down, depressed, or hopeless | ☐ | ☐ | ☐ | ☐ |
| 3 | Trouble falling or staying asleep, or sleeping too much | ☐ | ☐ | ☐ | ☐ |
| 4 | Feeling tired or having little energy | ☐ | ☐ | ☐ | ☐ |
| 5 | Poor appetite | ☐ | ☐ | ☐ | ☐ |
| 6 | Feeling bad about yourself – or that you are a failure or have let yourself or your family down | ☐ | ☐ | ☐ | ☐ |
| 7 | Trouble concentrating on things, such as reading the newspaper or watching television | ☐ | ☐ | ☐ | ☐ |
| 8 | Moving or speaking slowly that other people could have noticed. Or the opposite – being so fidgety or restless that you have been moving a lot more than usual | ☐ | ☐ | ☐ | ☐ |

| | | Not at all | Several days | More than half the days | Nearly every day |
|---|---|---|---|---|---|
| 9 | Thoughts that you would be better off dead, or hurting yourself in some way | ☐ | ☐ | ☐ | ☐ |

| GAD-7 | Over the last 2 weeks how often have you been bothered by any of the following problems? | Not at all | Several days | More than half the days | Nearly every day |
|---|---|---|---|---|---|
| 1 | Feeling nervous, anxious, or on edge | ☐ | ☐ | ☐ | ☐ |
| 2 | Not being able to stop or control worrying | ☐ | ☐ | ☐ | ☐ |
| 3 | Worrying too much about different things | ☐ | ☐ | ☐ | ☐ |
| 4 | Trouble relaxing | ☐ | ☐ | ☐ | ☐ |
| 5 | Being so restless that it's hard to sit still | ☐ | ☐ | ☐ | ☐ |
| 6 | Becoming easily annoyed or irritable | ☐ | ☐ | ☐ | ☐ |
| 7 | Feeling afraid as if something awful might happen | ☐ | ☐ | ☐ | ☐ |

| MOS-SSS | People sometimes look to others for companionship, assistance, or other types of support. How often is each of the following kinds of support available to you if you need it? | None of the time | A little of the time | Some of the time | Most of the time | All of the time |
|---|---|---|---|---|---|---|
| 1 | Someone to share your most private worries and fears with | ☐ | ☐ | ☐ | ☐ | ☐ |
| 2 | Someone to turn to for suggestions about how to deal with a personal problem | ☐ | ☐ | ☐ | ☐ | ☐ |
| 3 | Someone to do something enjoyable with | ☐ | ☐ | ☐ | ☐ | ☐ |
| 4 | Someone to love and make you feel wanted | ☐ | ☐ | ☐ | ☐ | ☐ |

Have you been told by a doctor that you have any of the following chronic health conditions?

1

☐ I have no chronic condition

| | | |
|---|---|---|
| ☐ Heart disease | ☐ High blood pressure | ☐ Leg pain when walking |
| ☐ Lung disease | ☐ Diabetes | ☐ Kidney disease |
| ☐ Liver disease | ☐ Problems caused by stroke | ☐ Disease of the nervous system |
| ☐ Cancer (within the last 5 yrs) | ☐ Anxiety Disorder | ☐ Depression |
| ☐ Arthritis | ☐ Substance abuse | ☐ Somatoform disorder |
| ☐ Personality disorder | ☐ Chronic pain disorder | ☐ Schizophrenic disorder |

Health status and prior treatment

1 Did you experience any episodes of depression/anxiety within the last year?

☐ I experienced no episodes
☐ I had one episode
☐ I had several episodes
☐ My symptoms of depression do not occur in episodes

During the last year, did you receive any of the following treatments for depression/anxiety?

| | | | | | |
|---|---|---|---|---|---|
| 2 | medication | ☐ no | ☐ 1-3 months | ☐ 3-6 months | ☐ more than 6 months |
| 3 | psychological treatment | ☐ no | ☐ 1-3 months | ☐ 3-6 months | ☐ more than 6 months |
| 4 | other | ☐ no | ☐ 1-3 months | ☐ 3-6 months | ☐ more than 6 months |

5 Has the treatment of your depression/anxiety over the last year been successful?

☐ very much ☐ moderately ☐ somewhat ☐ not at all

6 If you took any medication for depression/anxiety, did you take your medication as prescribed?

☐ ne ☐ mostly ☐ yes

Did you experience medication side-effects? ☐ yes ☐ no

7 If Yes, please indicate which side-effects you have experienced:

☐ Weight gain ☐ Sexual dysfunction ☐ Sleep disturbances

☐ Dry mouth ☐ Drowsiness/sedation ☐ Cardiovascular side-effects (e.g. palpitations)

☐ Gastrointestinal side-effects (e.g. diarrhea, nausea, vomiting) ☐ Other: ___________________________

8 How successful do you think your current therapy will be in reducing your symptoms?

☐ Not at all successful ☐ Somewhat successful

☐ Moderately successful ☐ Very successful

## Demographic factors

1 Which statement best describes your living arrangements?

☐ with partner/spouse/family/friends ☐ alone

☐ nursing home/hospital/long term care home ☐ other

2 What is your work status?

☐ Unable to work (due to a condition other than depression or anxiety) ☐ Unable to work (due to depression or anxiety)

☐ Not working by choice (student, retired, homemaker) ☐ Working part-time

☐ Seeking employment (I consider myself able to work but cannot find a job) ☐ Working full-time

3 How many working days have you missed within the last month due to illness? ________ (# of days)
