# Supplementary material for: Standardization of health outcomes assessment for depression and anxiety: recommendations from the ICHOM Depression and Anxiety Working Group
Source: Qual Life Res. 2017 Aug 7;26(12):3211–25. doi: 10.1007/s11136-017-1659-5 (PMC5681977; doi:10.1007/s11136-017-1659-5)
Supplement: Supplementary file 4 — Supplementary material 4 (PDF 446 kb) [file 11136_2017_1659_MOESM4_ESM.pdf]

ICHOM Treatment Monitoring for Depression and Anxiety v1

The following questions will assess your current health status helping your health care provider to monitor the treatment success and to acknowledge potential health risk factors.

PHQ-9	Over the last 2 weeks how often have you been bothered by any of the following problems?	Not at all	Several days	More than half the days	Nearly every day
1	Little interest or pleasure doing thing	☐	☐	☐	☐
2	Feeling down, depressed, or hopeless	☐	☐	☐	☐
3	Trouble falling or staying asleep, or sleeping too much	☐	☐	☐	☐
4	Feeling tired or having little energy	☐	☐	☐	☐
5	Poor appetite	☐	☐	☐	☐
6	Feeling bad about yourself – or that you are a failure or have let yourself or your family down	☐	☐	☐	☐
7	Trouble concentrating on things, such as reading the newspaper or watching television	☐	☐	☐	☐
8	Moving or speaking slowly that other people could have noticed. Or the opposite – being so fidgety or restless that you have been moving a lot more than usual	☐	☐	☐	☐
9	Thoughts that you would be better off dead, or hurting yourself in some way	☐	☐	☐	☐

GAD-7	Over the last 2 weeks how often have you been bothered by any of the following problems?	Not at all	Several days	More than half the days	Nearly every day
1	Feeling nervous, anxious, or on edge	☐	☐	☐	☐
2	Not being able to stop or control worrying	☐	☐	☐	☐
3	Worrying too much about different things	☐	☐	☐	☐
4	Trouble relaxing	☐	☐	☐	☐
5	Being so restless that it's hard to sit still	☐	☐	☐	☐
6	Becoming easily annoyed or irritable	☐	☐	☐	☐
7	Feeling afraid as if something awful might happen	☐	☐	☐	☐

		Not difficult at all	Somewhat difficult	Very difficult	Extremely difficult
1	If you checked off any problems, how difficult have these problems made it for you to do your work, take care of things at home, or get along with other people?	☐	☐	☐	☐

1 Did you receive any treatment for depression over the last two weeks?

medication	☐ no	☐ yes
psychological treatment	☐ no	☐ yes
other	☐ no	☐ yes
Did you experience medication side-effects?	☐ no	☐ yes

2 If Yes, please indicate which side-effects you have experienced:

☐ Weight gain ☐ Sexual dysfunction ☐ Sleep disturbances

☐ Dry mouth ☐ Drowsiness/sedation ☐ Cardiovascular side-effects (e.g. palpitations)

☐ Gastrointestinal side-effects (e.g. diarrhea, nausea, vomiting) ☐ Other: ______________________
